# Specialist to non-specialist teleconsultations in chronic respiratory disease management: A systematic review

**DOI:** 10.7189/jogh.11.04019

**Published:** 2021-03-27

**Authors:** Rutuja Patil, Rahul Shrivastava, Sanjay Juvekar, Brian McKinstry, Karen Fairhurst

**Affiliations:** 1 Vadu Rural Health Program, KEM Hospital Research Centre, Pune, India; 2 NIHR Global Health Research Unit on Respiratory Health (RESPIRE), Usher Institute, University of Edinburgh, Edinburgh, UK; 3 KEM Hospital Research Centre, Pune, India; 4 Savitribai Phule Pune University, Pune, India

## Abstract

**Background:**

Chronic respiratory diseases (CRD), are common public health problems with high prevalence, disability and mortality rates worldwide. Further uneven distribution of the health workforce is a major barrier to the effective diagnosis and treatment of CRDs. Teleconsultation between a specialist and non-specialist could possibly bridge the gap in access to health care and decrease CRD burden in remote areas. This review investigates the evidence for the effective use of specialist to non-specialist teleconsultation in the management of CRDs in remote areas and identifies instances of good practice and knowledge gaps.

**Methods:**

We searched for articles till November 2020, which focused on specialist to non-specialist teleconsultations for CRD diagnosis or management. Two independent reviewers conducted the title and abstract screening and extracted data from the selected papers and the quality was assessed by Joanna Briggs Institute’s (JBI) tool. A descriptive and narrative approach was used due to the heterogeneous nature of the selected studies.

**Results:**

We found 1715, articles that met the initial search criteria, but after excluding duplicates and non-eligible articles, we included 10 research articles of moderate quality. These articles were from nine different studies, all of which, except one, were conducted in high-income countries. The studies reported results in terms of impact on the patients, and the health care providers including primary care physicians (PCP) and specialists. The teleconsulting systems used in all the selected papers primarily used audio modes in addition to other modes like the audio-video medium. The included studies reported primarily non-clinical outcomes including effectiveness, feasibility, acceptability and usability of the teleconsultation systems and only three described the clinical outcomes. The teleconsultation was predominantly conducted in the PCP’s office with the specialist located remotely.

**Conclusions:**

We found relatively few, papers which explored specialist to non-specialist teleconsultation in management of CRDs, and no controlled trials. Two of the included papers described systems, which were used for other diseases in addition to the CRD. The available literature although not generalisable, encourages the use of specialist to non-specialist teleconsultation for diagnosis and management of CRDs.

The distribution of the health workforce across the globe is uneven and is often disproportionately lower in low and middle-income countries (LMICs) which are the areas often with the highest disease burden [1]. The World Health Organization (WHO)- Global Health Observatory reports that over 40% of member countries of WHO, all LMICs, have less than one physician per 1000 population [1]. The African Region suffers more than 24% of the global burden of all diseases but has access to only 3% of health workers and less than 1% of the world’s financial resources [1]. The unbalanced distribution of health care personnel by specialities adds to these disparities [2]. In India, while 69% of Indian population resides in rural areas and they are served by 22% of all health care providers compared with the 31% urban citizens who have access to 78% of trained health care professionals [3,4]. Poor access to transport and inadequate transport infrastructure in rural areas further aggravates the scarcity of health care workforce leaving a large proportion of the rural population without easy access to trained health care providers.

Information technology can facilitate access to specialist services, by overcoming the distance barrier by enabling specialist doctors to provide remote consultations to patients and provide support to general physicians and specialised local health workers [5,6]. Additionally, remote consultations have also provided opportunities for continued access to health care when maintaining social distancing during the global COVID-19 pandemic [7]. Telehealth care is thus one possible means of addressing the challenges related to uneven access to health care in India and globally [8].

Telehealth care is variously referred to as telemedicine, telehealth, telecare, telemonitoring, tele(discipline), teleconsultation and also eHealth in assorted contexts including mobile application based care, self-management with the help of remote monitors, doctor-to-patient remote consultation and also doctor-to-doctor consultation [9]. COCIR, the European Trade Association, representing the medical imaging, radiotherapy, health ICT and electromedical industries suggests a simpler definition that: “*Telemedicine includes all areas where medical or social data is being sent/exchanged between at least two remote locations, including both Caregiver-Patient/Citizen as well as Doctor to Doctor communication”* [5]. This definition is most applicable where telemedicine seems to have widened its scope to a variety of health information exchange for managing health and well-being of the population. For this systematic review, we will refer to this as 'Teleconsultation’.

Chronic respiratory diseases (CRD) especially asthma & Chronic Obstructive Pulmonary Disease (COPD) are common public health problems with high prevalence, disability and mortality rates worldwide [10-13]. Asthma is the 14^th^ most important disorder in the world in terms of the disease burden [14], and COPD is the third leading cause of death, worldwide [15]. Diagnosis of these chronic respiratory diseases is based on detailed patient history and clinical assessment, aided by specialised tests such as spirometry and peak expiratory flow readings. However, interpreting the results of such tests require specialised training [16]. Specialists are not always available in rural regions worldwide. A study conducted in the US reported that less than one-third of the rural population, but nearly 98% of the urban American population had access to a pulmonologist within a 10-mile radius [17]. The situation in low- and middle-income countries (LMICs) like India is comparatively worse. The Association of Chest Physicians of India lists 2413 chest physicians in a country of 1.3 billion people, more than 95% of these are registered at in large urban areas [18] as compared to American Thoracic Society comprising of more than 16 000 registered members including physicians, researchers, advanced practice nurses, respiratory therapists which serves nearly 331 million population [19]. The rural areas in LMICs are primarily served by primary care physicians (PCP) or allied health staff including Auxiliary Nurse Midwives (ANMs) nurses or pharmacists for delivery of primary health care [20]. Lack of access to pulmonologists and other specialists required for the diagnosis and management of the CRDs may be one of the factors contributing to the misclassification or delayed diagnosis of CRDs and inappropriate disease management. Use of teleconsultation to remotely support the PCPs by engaging specialists could help in scaling up CRD diagnosis and treatment.

The objective of this systematic review was to explore the evidence for the effective use of specialist to non-specialist teleconsultation in the management of CRDs in adults in remote areas and to document best practices and challenges; and identify, knowledge gaps and provide recommendations for future research.

## METHODS

### Search strategy

A search strategy tool SPIDER (sample, the phenomenon of interest, design, evaluation, research type) [21] was used to design the search strategy. We consulted a librarian when creating the search strategy. The Search terms included variations of “Telehealth”, “Telehealthcare”, “eHealth”, “Remote consultation”, “Satellite consultation”, “Remote diagnosis”, “Remote management”, “Teleconsultation”, “Tele- opinion”, “Tele- diagnosis”, “Remote medical consultation”, “Telemedicine”, “Primary care”, “Remote clinic”, “Satellite clinic”, “videoconference”, variations of “Chronic Respiratory diseases”, “Chronic obstructive pulmonary disease”, “asthma”, “Face to face consultation”, “doctor to doctor consultation”, “physician to physician”, “referral”, “consultation” (Appendix S1 in the **Online Supplementary Document**).

### Database

We used *Embase*, *Medline* (through Ovid), *PubMed*, and CAB *Global Health* to run our search terms. RP hand-searched citation lists to identify potentially relevant papers within retrieved papers and review articles.

### Search duration

We searched for papers up to November 2019 and later the search was repeated in November 2020. We did not restrict the start date, although first publication in the search appeared in 1975.

### Inclusion and exclusion criteria

We considered all types of research methodologies including interventions as well as observational studies. All types of teleconsultation between specialists and non-specialists for CRD diagnosis or management, including but not limited to email, videoconference, telephone etc. were included. We excluded articles, which were focused on home-based care, mobile health, doctor to patient consultations, molecular outcomes, pulmonary rehabilitation, telephone surveys, post-hospitalisation follow-ups, telemonitoring and tele-training. We also excluded reviews, systematic reviews and conference abstracts and the search was not restricted to any particular language.

### Data extraction and analysis

The title and abstract screening followed by data extraction from the selected papers was independently conducted by two reviewers (RP and RS). The search results from all databases were imported into Mendeley (1.19.5) [22] and the duplicates were removed. The titles and abstracts were screened for all the papers. We selected studies mainly on two criteria. The first criteria is the disease condition, including at least one chronic respiratory illness, and the second criteria is consultation type, where a specialist provided consultation to non-specialists or a PCP, to either diagnose or treat the chronic respiratory disease.

Data extraction into Microsoft excel was developed based on the STROBE [23], NICE [24], CASP [25] and CONSORT [26] guidelines and included fields on the type and aim of the study, population description, disease condition, type of consultation, type of outcome and facility. The discrepancies were resolved by discussions between RP and RS. The narrative data was extracted using summary tables independently by two authors (RP and RS) which were later combined by one reviewer (RP) into one sheet and all authors reviewed this sheet regularly. Mendeley (1.19.5) [22] was used to manage references and MS-Excel (Microsoft Inc, Seattle, WA, USA) was used for data management. The quality assessment of the papers was carried out using the Joanna Briggs Institute’s (JBI) tool for critical appraisal [27] by two reviewers (RP and RS). The discrepancies were resolved by discussion.

The study designs of the selected papers were heterogeneous thus a narrative approach to data synthesis was used rather than a meta-analysis.

## RESULTS

**Figure 1:**
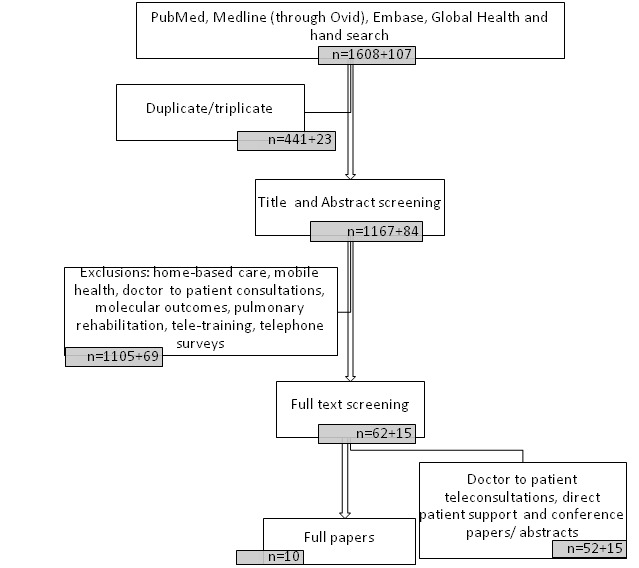
PRISMA-flow diagram for the selection of papers.

The identified papers, the screening process, and the final number of studies included are detailed in the PRISMA flowchart (**Figure 1**). In summary, we found 1715 papers in the above-mentioned databases, of which 464 were duplicates, which were excluded, leaving 1251 papers. We screened the abstracts of all these 1251 papers of which 77 were selected for full-text screening based on the inclusion criteria. We further selected only published papers hence we did not include 21 meeting abstracts/conference papers. We found 10 relevant papers which included all three parameters of (i) teleconsultation, (ii) Specialist to non-specialist consultation (iii) CRDs.

### Study population

The study populations in the selected studies primarily included two groups namely the patients and the health care provider including PCPs and specialists. Some studies also conducted secondary data analysis thus indirectly including patients as study participants [28,29]. The demographic characteristics of the study population were not described in any of the papers. The available details including a description of the recruitment methods in the included studies are described in **Table 1**.

**Table 1:** Study population and selection criteria

Report title	Population description	Recruitment methods	Sample size	Sampling details/ technique
Koizumi et al [30]	Patients: The subjects were two patients with chronic obstructive pulmonary disease (COPD) receiving home oxygen treatment. Healthcare providers: Specialists at tertiary care hospital and primary care doctors from the local hospitals	The patients were selected after their treatment policy was decided which included home oxygen treatment. This treatment was assigned to the local hospital doctor through regular hospital visits who shared the patient’s information with the specialist	Patients: 2 Chronic Obstructive Pulmonary Disease (COPD) patients. Healthcare providers:	Purposive who received treatment at Shinshu University Hospital
Raza et al [28]	Patients: the patient at the Iron Mountain clinic Healthcare providers: Pulmonary specialist at the Milwaukee site and the ancillary provider (nurse or respiratory technologist - RT) at the Iron Mountain.	Pulmonary specialist categorized the patient referral in urgent and non-urgent. The Non-urgent patients were eligible for teleconsultation.	Patients: 314 patients in 684 clinical encounters	All patients presenting at the spoke site were interviewed by Nurse for complaint medication and diagnostics if any. All patients who received pulmonology teleconsultation during the study period of seven years. Only non-urgent patients were considered suitable for telemedicine. The program provides outpatient teleconsultations (pulmonary, rheumatology, endocrine, mental health, and infectious disease). However, the paper presents data on pulmonary teleconsultations from one spoke site located 354kms away from the hub.
Averame et al [31]	Patients: requiring telespirometry. Healthcare providers: Primary Care Physicians (PCP)s and pulmonary specialist.	PCPs from all regions of Italy who were willing to participate and perform the spirometry were involved. Fifty-one pulmonary units distributed throughout the country were asked to participate in the study. However the methods do not describe how the participants were approached	Patients: 9312 patients. Healthcare providers: 638 PCPs 51 pulmonologist.	Not mentioned for PCPs and pulmonology units. The participants were enrolled by the PCPs from his/her general practice in the following categories of subjects: (1) current or ex-smokers without respiratory symptoms; (2) subjects with respiratory symptoms (3) subjects with a pre-existing clinical diagnosis of bronchial asthma; and (4) subjects with a pre-existing clinical diagnosis of COPD.
Bonavia et al [32]	Patients: requiring telespirometry. Healthcare providers: PCPs and pulmonary specialist.	PCPs from all regions of Italy who were willing to participate were involved	Patients: 17910 patients. Healthcare providers: 937 PCPs trained by 51 pulmonologists.	PCPs who were willing to conduct telespirometry. Pulmonology units identified by a steering committee. Subjects on whom the PCP conducts telespirometry. The paper mentions that the details are described in another publication describing this study [31] however, no more details are mentioned about the recruitment methods
Bernocchi et al [33]	Patients: Patients who were screened for eligibility before hospital discharge and who consented.	Patients at the PCP clinic. Before discharge from the hospital all patients were given instruction on their respective disease conditions (CHF or COPD). Patients were given pointers for recognizing signs and symptoms of decompensation/worsening. Patients with CHF were provided with a portable 1-lead electrocardiogram (ECG) device (Card-Guard 2206). Patients with COPD received a pulse oximeter (Vitalaire, Italy) device	Patients: 4925 patients	Not mentioned
	Healthcare providers: PCPs and pulmonary specialist		Healthcare providers:	
Thijssing et al [34]	Patients: Patients who were considered suitable for the telepulmonology consultations by the PCP. Healthcare providers: PCPs and pulmonary specialist	All PCPs registered in the Netherlands and in possession of a spirometer that could be linked to a computer were eligible. PCPs opted in to join the teleconsultation system	Patients: 1958 tele pulmonology from 1828 patients. Healthcare providers: 158 PCPs and 32 pulmonologists.	All PCPs registered in the Netherlands and in possession of a spirometer that could be linked to a computer. Participating PCPs decided on suitability of tele pulmonology consultation. All telepulmonology conducted during the study period were included.
Metting et al [35]	Patients: Patients suspected to have obstructive airway disease (OAD) Healthcare providers: PCPs and pulmonary specialist	Patients are decided by PCPs. All PCPs in study area. Pulmonologist who has assessed more than 300 patients were approached for the service	Patients: 11401 patients Healthcare providers: 360 PCPs and 10 pulmonologists	Not mentioned.
Fadaizadeh et al [36]	Patients: Thoracic surgery patients who consented for teleconsultation Healthcare providers: Physicians and nurses at the hospital and Consulting physician at remote location	Patients selected for teleconsultation were mostly complicated thoracic surgery cases with multiple organ failure for whom transportation was not only infeasible, but also contraindicated. Physician located in remote hospital and long distance travel was required to perform visit	Patients: 58 patients. Healthcare providers: one consulting physician	All patients who consented to participate in the study during the study period
Weikert et al [37]	Secondary data analysis no study population	Partner hospitals located in Central Eastern Europe and Asia were eligible to participate in the cross-border teleradiology program for a structured expert consensus reading of CTs with the clinical question of IPF. Partner hospitals located in Central Eastern Europe and Asia were eligible to participate in the cross-border teleradiology program for a structured expert consensus reading of CTs with the clinical question of IPF.	703 HRCT scans Data from 239 hospitals located in 46 cities in 12 countries	Not mentioned
Wrenn et al [29]	Patients: Patients attending the service and who completed the teleconsultation. Healthcare providers: PCP from eight adult primary care sites at the University of California, San Francisco (UCSF), USA	First 200 teleconsultations during initial months of program	Patients: 195 patients. Healthcare providers: 86 PCP	Not mentioned

### Consultation type and facilities used

We included studies where a specialist communicated with a non-specialist by teleconsultation for either diagnosis or management of the patient’s condition. In one study, consultations were conducted in real-time [30], the others were stored and forwarded consultations, which meant the specialists were sent the patient details and reports by the non-specialists and the specialists provided their opinion to the non-specialists within a stipulated time frame.

The case studies conducted in Japan described a setup which comprised of equipment at three locations which included the home of two patients with chronic respiratory failure, the hospital of the attending PCP, and the hospital of the pulmonary specialist [30]. No other study reported home-based specialist to non-specialist teleconsultation.

The primary care centres or PCP’s offices were used as the site of the teleconsultation in six studies which were reported in seven papers [28,29,31-35] where the consulting specialist was located at a remote location. These studies could be categorised as the hub and spoke where multiple PCPs were provided with an opinion by specialists for either diagnosis or management of CRDs. In one study reported from the USA, the hub was located at Milwaukee Veteran Affairs Medical Centre (VAMC) pulmonary telemedicine clinic which was located away from the spoke centre at Iron Mountain VAMC [28]. Five other studies had similar approach where the teleconsultation was conducted at the PCP’s office and the specialist provided an opinion for the diagnosis or management remotely [29,31,32,34,35]. The teleconsultation was conducted between specialists at local hospitals and specialists at a tertiary care hospital in two studies [36,37] (**Table 2**).

**Table 2:** Study characteristics

Report title	Year of publication	Country in which the study is conducted	Type of study	Disease condition	Consultation type
Koizumi et al [30]	2005	Shinshu University Hospital, Japan	Case study	COPD	Real-time communication between patient at home, attending primary care physicians (PCP) at hospital and pulmonary specialist at another hospital once a week.
Raza et al [28]	2009	United States	Retrospective analysis for description of a telepulmonology clinic	Pulmonary conditions	Pulmonary Specialist to Nurse or respiratory technologist
Averame et al [31]	2009	Italy	Observational study	Asthma and COPD	Training to PCPs by pulmonologists followed by PCPs performing spirometry in their clinic which was checked for quality by the gen at the central office providing teleconsultation and further offered an interpretation of the results which was faxed to the PCPs’ office.
Bonavia et al [32]	2009	Italy	Observational study	persistent respiratory symptoms, or a previous diagnosis of asthma or COPD	Training to PCPs by pulmonologists followed by PCPs performing spirometry in their clinic which was checked for quality by the specialist at the central office and further offered an interpretation of the results which was faxed to the PCPs’ office.
Bernocchi et al [33]	2012	Italy	Description and assessment of an ongoing project	Chronic Heart Failure, Skin, Diabetes, Chronic Obstructive Pulmonary Disease	Second opinion for the PCPs in Cardiology, Dermatology, diabetology and pulmonology
Thijssing et al [34]	2014	Netherlands	Intervention study	COPD	Pulmonologists to PCPs
Metting et al [35]	2015	Netherland	A cross-sectional baseline description and longitudinal results	Asthma, COPD and overlap syndrome - obstructive airway disease	PCPs refer patients to service and tests are conducted in laboratory. Pulmonologists inspect the data through the internet and send the PCP diagnosis and management advice
Wrenn et al [29]	2017	USA	A retrospective descriptive analysis	Cardiology, Endocrinology, Gastroenterology/hepatology, Haematology, Infectious diseases, Nephrology, Pulmonary medicine, Rheumatology	Specialist provided teleconsultation to patients via the PCPs
Fadaizadeh et al [36]	2018	Iran	Cross-sectional Interventional	Patients who have indergone thoracic surgery with multiple organ failure	Pulmonologists to PCPs
Weikert et al [37]	2019	Partner hospitals in twelve Central Eastern Europe and Asia from the collaboration framework with Boehringer Ingelheim	Description and analysis of Intervention	presence of Usual Interstitial Pneumonia (UIP)-pattern in patients with suspected Idiopathic Pulmonary Fibrosis	Radiologist and a pulmonologist reported the diagnosis of HRCTs to another pulmonologist or a referring physician

### Details of the systems used for teleconsultations

The specific teleconsultation systems used in all the selected papers varied; broadly they primarily used audio modes but some use a mixture of audio-video. Three papers from two studies focused on diagnosis using telespirometry.

Koizumi et al. shared clinical information, using a remote biological information transmission device which was installed at the patient’s home. Equipment to measure arterial blood oxygen saturation and blood pressure was set up on the patient’s finger along with three electrodes for Electrocardiogram. The remote consultation management device was set up at the attending PCP's hospital. Besides, another device for consultation management and monitoring was set up at Shinshu University Hospital, where the pulmonary specialist was located [30].

Another study described a hub-and-spoke model where the nurse or respiratory therapist (RT) at the spoke site recorded the clinical history and conducted a focused pulmonary physical examination. Medical consultations at the hub site were conducted via the live, two-way audio and video conferencing system. The physician at the hub site viewed chart notes and images of Electrocardiogram and radiographs and further prescribed diagnostics, medicines and suggested if the patient needed face to face consultations [28].

The Italian Alliance study published two papers in 2009 focusing on telespirometry for diagnosis of CRDs. One pulmonary specialist provided education and training on spirometry and diagnosis of obstructive airway diseases, to a group of PCPs in a six-hour education session. Each PCP was equipped with a simple, portable pneumotachograph (Spirotel, MIR, Roma, Italy) to measure the main indices derived from a maximal forced expiratory manoeuvre, the reports of which were transmitted to a central office. The operator at central office established a real-time communication and commented on the quality of the spirometry traces, invited the PCP to perform additional expiratory manoeuvres in the same subjects, and offered an interpretation of the results of the single patient. Then, the report was sent by fax to the PCP’s office [31,32].

The descriptive paper on the TELEMACO project narrates that a service centre provided support to PCPs during daily in-office or home visits and for pulmonology consultations, PCPs contacted the service centre using a real-time telephone only and no special service was used [33].

An intervention study focused on telepulmonology consultations used a hypertext transfer protocol secured, web-based teleconsultation system for communication between PCP and pulmonary specialist. PCPs accessed a secured web-based teleconsultation system (KSYOS Telemedical Centre, Amstelveen, The Netherlands) where they completed the patient personal data, added up to four PDF’s of the spirometry results and optionally added additional relevant clinical information. This information was sent to the local pulmonary specialist who had to answer within two working days [34].

Another study which focused on providing asthma and COPD services used an Electronic diagnostic support (EDS) system. After referral of a patient by the PCP to the asthma COPD services, a trained lung function technician conducted spirometry and along with the patient completed the patient information and clinical history in the EDS. This information was accessed by a Pulmonary specialist for assessment within five days which was then reported to the PCP who further decided on disease management [35].

A similar store and forward system was used by Fadaizadeh et al. for patients who have undergone thoracic surgery with multiple organ failure. The receiving PCP provided all necessary documents (the patient’s history, tests, ECG, radiology documents) and transfer to the specialist via store and forward, and then discussed the case online via videoconference. Fibre optic communication; and web conference software was used for a simultaneous audio-visual connection. Tele-examination devices and equipment were provided including a camera for examining the ear, eye and also digital stethoscope for heart and lung examination. A communication network for specialised consultations was established among seven specialised hospitals [36].

The study which described the use of High-Resolution Computed Tomography (HRCT) scans for identifying Usual Interstitial Pneumonia (UIP)-pattern in patients with suspected Idiopathic Pulmonary Fibrosis with clinicians performing the Chest HRCT scans locally at the referring hospitals. The HRCT scans were sent for a centralised expert consensus reading by a radiologist and a pulmonary specialist. After registration, a pulmonary specialist in partner hospitals received a personalised upload link valid for a single patient who had consented and for short period. All data for the patient were uploaded to the central expert reading site. A radiologist and pulmonary specialist together generated report for the patients and the reports were sent back by encrypted email to the referring PCP in the local hospital [37].

A retrospectively described study used eConsult services, where the PCPs at the point of referral, had the option to submit an eConsult request, using a structured template, if they believed the specialist could address the clinical question without an in-person evaluation. Specific structured referral templates for the most common clinical problems referred to each speciality, which incorporated relevant laboratory data from the EHR, allowed PCPs to identify if relevant imaging data were available, and asked the PCP to provide a recent assessment and a specific clinical question [29].

### Study outcomes

The included studies focused primarily on non-clinical outcomes including effectiveness, feasibility, acceptability and usability of the teleconsultation systems. Three of the 10 studies described the clinical outcomes more clearly [28,35,37] one study which described observations of patients’ assessment as clinical outcomes [30] did not provide sufficient details to assess their methods. The description of the study methods used to collect the data and respective study outcomes are listed in **Table 3**. We further describe the common conclusions from all the 10 selected papers.

**Table 3:** Methods and outcome

Report title	Methods for data collection	Study duration	Analysis	Clinical outcomes	Other outcomes	Study limitations and critique
Koizumi et al [30]	Attending PCPs took the medical history. Patients measured the Blood Pressure, Electrocardiogram and heartrate and oxygen saturation which was recorded in the system and partial pressure of expiratory carbon dioxide was reported to the physician verbally.	One year for patient 1 and for patient 2 not mentioned. Weekly communication within all three parties	No analysis is presented in the paper	1. Depression; 2. Other health issues; 3. Respiratory symptoms were observed and discussed; however, the paper does not mention clear clinical outcomes neither the methods which were used for measuring these clinical outcomes	Effectiveness of system connecting multiterminal. The trial program resulted in the same information being exchanged remotely using the multi-station teleconsultation system that would be exchanged in a direct, face to-face encounter	The clinician’s observations are described in the results section. No specific method is mentioned to document the observations. The paper does not clearly mention the aims of the publication. The result describes that the system is effective yet the methods do not describe measuring the effectiveness although the study of two cases proves the feasibility of the intervention.
Raza et al [28]	Retrospective data analysis. The authors extracted data from computerized patient record system, paper chart, physician logbooks of teleconsultation visits, patients written comments after completion of teleconsultations.	January 1998 and December 2004 (7 y)	Descriptive statistics were undertaken for the study outcomes. Data analysis was done using Microsoft access, SPSS and ArcGIS. Analysis for patient satisfaction data are not described.	1. Reasons for referral and access to specialty care: Common reasons- abnormal thoracic radiography (38%), COPD (26%), and work-up of dyspnea (13%); 2. Medical process of care: Due to limited scope of physical examination, consultant, relied on the exam findings documented by the nurse or respiratory therapist present with the patient (vital signs documented in 99.7%, physical examination in 84% and respiratory examination in 92% patients); 3.Physician diagnosis: COPD (29%), benign pulmonary nodule (11%), bronchial asthma (6%), and lung cancer (6%). Final diagnosis by physicians after first consultation in 90% cases.	1. Medical decision making: clinically significant change in management for 41%) patients and follow-up required in 51% patients. 8% required face to face visit t the hub. 2. Study population and travel distances: Total travel distance and time saved for 684 visits is 473 340km or 294 120 miles and 748 work days considering only patient would have travelled to the hub and no one accompanied the patient. 3. patient satisfaction: Overall positive experience	The data analysed is extracted from the system included a range of sub-specialities however this paper describes data from pulmonary patients only. The analysis for the qualitative data are not described. Data from single teleconsultation spoke centre is described which cannot necessarily be representative of the complete system. There is an assumption that all encounters would have resulted in long distance referral, reducing the bar to consultation may have increased demand a historical comparator might have been useful
Averame et al [31]	The PCPs performed spirometry in their office after the training which was sent to specialist at the central office, the criteria used to accept a single telespirometric test were the presence of two at least acceptable and reproducible manoeuvres. Fixed diagnostic criteria was considered for interpretation of results. FEV1/FVC fixed ratio, and not FEV1/VC% predicted, was used to assess for the presence of airway obstruction. Patient characteristics were collected in case report form by the PCPs.	Not mentioned in the paper	Descriptive statistics, consisting of numbers and percentages for ordinal and categorical variables and means with standard deviations or medians with ranges for continuous variables and valid cases, are presented	Not mentioned	78% (7262) of the telespirometric tests were classified as acceptable of these 7262, 41% (3003) exhibited abnormal spirometric pattern. 23% of patients had a clinical diagnosis of COPD but normal telespirometry. Subgroup analysis was done for smoker, non-smokers, symptomatic, already detected COPD and Asthma.	Although the training provided to the PCPs is mentioned in the methods, the training assessment is not provided in the results. The distribution of 22% unacceptable spirometries are not described, if these were from a set of specific PCPs or dispersed amongst all 638 PCPs. The study participants were selected group of participants as selected by the PCPs and not a random representation of the population. The methods describe that the five pulmonology units provided trainings the number of specialists who supported the PCPs in assessing the spirometries is not mentioned.
Bonavia et al [32]	Telespirometry was conducted by the process as mentioned in the previous publication of the same study (Averame et al) [31]. Further a turbine spirometer (Spirotel) was use to perform these spirometries (not mentioned in the Averame et al). The data for comparison between Spirotel and conventional laboratory spirometry is not presented in these papers. Quality evaluation of the telespirometries was done by two authors based on ATS recommendation and office spirometry.	October 2002 and ended in October 2004	Descriptive analysis was performed (number of observations, means and standard deviations, and categorical data as absolute and percent frequencies.)		22.2 ± 25.2 tests per PCP during study period. 70% of the tests met the criteria for good or partial co-operation, allowing spirometric abnormalities to be detected. Normal telespirometry (38.9); Mild airway obstruction (4.5); Moderate airway obstruction (9.5); Severe airway obstruction (4.5);Very severe airway obstruction (0.7); Abnormal spirometry, but not clear airway obstruction (8.5); Suspected airway obstruction (3.3)	The number of PCPs sending at least one spirometry per month reduced to less than 100 by end of the study. This shows that there were problems in continuity of the telespirometry services especially in access to TCO and PCPs time for conducting telespirometry. Although these explanations are discussed in the discussion, the methods do not mention how these were confirmed. The aim of the study is to determine feasibility yet the methods do not mention measuring the hurdles or facilitators in conducting the telespirometry. Only telespirometry were assessed for acceptability and cooperation.
Bernocchi et al [33]	Assessment of TELEMACO network: By evaluating the organization of the integrated services across the Lombardy territories. Acceptance of TELEMACO Services by the Health Authority: By determining whether a system of reimbursement for the services provided could be implemented by the regional authorities after the project was completed	6 mo	Descriptive statistics	No consultation was used for pulmonology in the component 2 (component 2 is relevant to the objectives of the systematic review) of the system.	Assessment of TELEMACO network: Implementation of project had positive impact on innovations in working methods and procedures. Acceptance of TELEMACO Services by the Health Authority: health authority decided to implement new health networks for better home care	The study has three components, of which only second component which describes “Second opinion for PCPs in cardiology, Dermatology, Diabetology and pulmonology” is included in the description of the study for purpose of this systematic review. Moreover, the methods to assess the network and the acceptance are not described in details in the paper.
Thijssing et al [34]	1. Quality of Care: (using a questionnaire completed by PCPs after the TPCs) The % of Telepulmonology Consultation (TPC) requests, the %of patients physically referred by PCPs to pulmonologists, education effect experienced by PCP, % of TPCs in which the PCP was helped with the pulmonologist’s response and mean response time of pulmonologists; 2. Efficiency of Care: % of prevented physical referrals, % of physical referrals who otherwise would not have been referred to a pulmonologist.	April 2009 and November 2012. (3 y and 8 mo)	Descriptive statistics	Twenty-three percent of the patients were diagnosed with COPD by pulmonologist. The pulmonologist answered ‘Unsure’ to the question ’Diagnosis COPD?’ in 16% and with ‘No’ in 61% of the cases.	Quality of care: 1. percentage of TPCs sent by PCPs for which advice of a pulmonologist was requested: 69%; 2. 92% PCPs admitted to have learned from the pulmonologists; 3. in 96% cases, the PCPs or patients found the pulmonologists' advice helpful; 4. percentage of patients physically referred by the PCP to the pulmonologist, who would not have been referred without telepulmonology: 18% of the TPCs; 5. Pulmonologists answered the TPCs on average after 18.2 working hours. Efficiency of care: 1. the percentage of prevented physical referrals: 27%	Telepulmonology can contribute to more efficiency and a higher quality of care for COPD patients. The clinical follow up of the patients is not done, neither any data mentions if the patient visited the pulmonologists after the suggested referral. The pulmonologists are not asked about the services/ the data are not presented in the paper. The pulmonologists responded after 18 h average; however, the paper does not mention how the patients were informed about the diagnosis/ treatment suggested.
Metting et al [35]	1. Feasibility: the proportion of PCPs in the target area who used the AC service, the proportion of patients with asthma or COPD who were assessed by the service, the quality of the spirometry and the number of patients that could be diagnosed, and the variation in diagnostic pattern between the different pulmonologists by using χ^2^. 2. Follow-up visits. Patients for whom medication change was advised by the pulmonologist were automatically scheduled for an additional follow- up assessment after 3 mo	2007 to 2012 (5 y)	Nonparametric paired tests were used to compare baseline data with follow-up data. Paired *t* tests were used for the longitudinal evaluation of FEV1(in litres). Follow-up data of baseline GOLD stages are presented to show the distribution of these patients to other GOLD stages	1. Usable quality for diagnosis- spirometry as per pulmonologist: 93.6%; 2. diagnosed patients: 79.4%; 3. Baseline diagnosis matching with follow-up: 91.2%; 4. Inhalation technique improved significantly: from 35.1% to 52.5% in three months; 5. Significant improvement in Asthma and COPD status during follow-up: Well controlled asthma patients from 23.9% to 49.5% and well controlled COPD patients from 27.4% to 48.9% in three months	79.3% of the PCPs in the target area referred patients to the service. 60% of adults’ asthma and COPD patients were referred at least once.	If PCP followed the referral recommendation of the pulmonologist is not documented
Fadaizadeh et al [36]	(1) Compare the pace of tele-consultation and regular (bedside) consultation: Patient records assessed and the mean time between requesting consultation and visit by the off-site physicians was evaluated and compared; (2) Physicians’ satisfaction from tele-Consultation: A questionnaire comprising of three choices was used (fully satisfied, partly satisfied, and not satisfied)	October 13 to December 2015 (26 mo)	Comparison of mean time using Mann- Whitney non- parametric test.	The highest rate of consultation was in neurology (27 cases) and thereafter, in neurosurgery (11 cases). Other consultations were in endocrinology (3 cases), gastroenterology (2 cases), thoracic surgery (2 cases), vascular surgery (2 cases), ophthalmology (1 case), haematology (1 case) and dermatology (1 case)	1. Teleconsultations were answered 2.5 times faster than face to face consultation; 2. Satisfaction survey results showed that the physicians were fully satisfied with teleconsultations in 82.75% of cases. They were partly satisfied in 12.06% and not satisfied in 5.17% of consultations	The paper describes the tele-ICU for thoracic surgery patients however, the consultation provided was mostly for other problems and only two consultations of thoracic surgery are mentioned in the results. Moreover, the number of consulting physician/s is not mentioned in the paper
Weikert et al [37]	Authors evaluated basic patient characteristics (age and sex) as well as the geographic distribution of referring hospitals. Furthermore, authors analysed technical aspects like slice thickness, tube current (mAs) and peak kilovoltage, determined whether the slice thickness of transmitted CTs complied with the recommendations defined by the Fleischner Society and in the ATS/ERS/JRS/ALAT-guideline. A questionnaire based online survey was conducted to assess satisfaction with and impact of the program and the structured reports that were generated within the context of the teleradiology program.	Jan 2014 to May 2019 (5 y)	Descriptive statistical analysis	Satisfaction with the centralized IPF expert teleradiology program was 8.4 (out of 10). Their impact on the clinical management of the patients was rated 9.0/10. The utility of the teleradiology program regarding the gaining of own expertise in IPF was assessed as 9.3/10.	All referring physicians (100%) stated that they would recommend the centralized IPF teleradiology program to their colleagues	The HRCT referral although was intended from 12 countries, half of those did not contribute to even 10% of the total number of scans. The survey was taken only by one third of the total participating physicians hence the satisfaction results couldn’t be generalized.
Wrenn et al [29]	Authors categorized the question asked during teleconsultation as “diagnosis,” “treatment,” and/or “monitoring.” They further reviewed the medical record to determine the percentage of specialist recommendations PCPs implemented, and the proportion of patients with a specialist visit in the same specialty as the teleconsultation emergency department visit, or hospital admission during the follow-up	August 2012 and January 2013 (6 mo)	Descriptive statistics	No clinical outcomes	1. PCPs asked questions related to diagnosis in 71% of cases, treatment in 46% of cases, and monitoring in 21% of cases; 2. CPs ordered 79% of all recommended laboratory tests, 86% of recommended imaging tests and procedures, 65% of recommended new medications, and 73% of recommended medication changes. In the six months after the teleconsultation, 14% of patients had a specialist visit within the UCSF system in the same specialty as the teleconsultation	The patient visit to specialists were recorded if the patient visited the same hospital, however, there is a possibility that the patients could access another health care facility. The results are analysed from the data collected from one centre hence the results may not be generalizable.

#### Effectiveness

Two papers described the effectiveness of the existing system. Koizumi et al. describes that the system was effective for establishment of appropriate treatment, provided the cooperation between the pulmonary specialist and attending PCP was established and further suggests that a similar system could be considered useful and promising for further use [30]. Fadaizadeh et al. studied teleconsultation between physicians and nurses at the thoracic surgery hospital and consulting physicians, in thoracic surgery patients in intensive care unit and concluded that teleconsultation improved decision-making in thoracic surgery ICU patients through time saving and accelerating off-site consultations [36] (**Table 4**).

**Table 4:** Study aim and outcomes

Report title	Study population description	Aim of study	Type of outcome measure	Facility description
Koizumi et al [30]	Two patients with chronic respiratory failure, attending PCP, and one pulmonary specialist	To create and test a multi-station teleconsultation support system, three remote locations were connected: the homes of two patients with chronic respiratory failure, the hospital of the attending PCP and a pulmonologist in another hospital	The effectiveness of a system connecting multiple terminals for teleconsultation. (Notes: although the methods to measure the effectiveness is not described). The feasibility of use is established	Two patients with chronic respiratory failure, the hospital of the attending PCP, and the hospital of the pulmonary specialist.
Raza et al [28]	Six pulmonary specialist at the Milwaukee site and the patient and ancillary provider (nurse or respiratory technologist - RT) at the Iron Mountain. 314 patients	The goals of the study were (1) to evaluate the use and effect of teleconsultation technology to provide consultative outpatient care for a broad range of pulmonary conditions; and (2) To evaluate the use of a teleconsultation program in terms of (a) access to care (including reduction in both travel for patients and waiting time for appointments), (b) clinical decision making (medical interview and physical exam, medical work-up required, and outcome of teleconsultation), and (c) patient disposition (need for follow-up care and need for in- person evaluation)	1. Reasons for referrals and access to speciality care; 2. Medical process of care 3. Physicians’ diagnoses after teleconsultations 4. Medical decision making 5. Patient demographics 6. Patient Satisfaction. 7. Number of visits and Travel distances	Remote location (Spoke): Iron Mountain VAMC in Iron Mountain, MI and Provider (Hub): Milwaukee Veteran Affairs Medical Centre (VAMC) pulmonary teleconsultation clinic
Averame et al [31]	638 PCP and pulmonary specialist for interpreting the telespirometry	To report feasibility and usefulness of telespirometry in general practice from Italian Alliance study. (Aim of the Alliance study was (a) to improve the familiarity of PCPs with spirometry; and (b) to demonstrate the usefulness of a spirometric evaluation)	Diagnostic accuracy of PCPs interpreting spirometry in asthma and COPD according to ACCP, GOLD and ATS/ERS Guidelines	PCP office and pulmonary units
Bonavia et al [32]	large sample (937) of PCPs from all regions of Italy and Fifty-one pulmonary units distributed throughout the country. 17 910 subjects	To report the results of an Italian study on the feasibility of telespirometry in general practice	Feasibility of telespirometry in general practice	PCP office and pulmonary units
Bernocchi et al [33]	Patients, 176 PCPs two specialists	Combining management, clinical, and technological tools to improve provision of health care in rural areas, from secondary care community hospital settings to home care	The aims of the project were 3-fold: (1) to implement and use continuity-of-care services (2) to design a network in the territory for sharing continuity-of-care programs for the management of chronic diseases (3) to allow the health authority to collect data to establish sustainable pricing at the regional health level for implementing TM	1. Home-Based Tele management for Patients with Chronic Heart Failure or Chronic Obstructive Pulmonary Disease 2. Second opinion for PCPs at PCPs’ Office 3. Second opinion on digital images between PCPs office and specialist hospitals.
Thijssing et al [34]	158 PCPs and 32 pulmonologists	To assess the effect of telepulmonology on quality and efficiency of care	Effect of telepulmonology on quality and efficiency of care	PCPs office and pulmonary specialist’s office
Metting et al [35]	11401 patients suspected to have obstructive airway disease (OAD) 360 PCPs from north Netherland, 10 pulmonologists	To improve the management of asthma and COPD patients in primary care using an internet-based service	Feasibility, effectiveness and efficiency in supporting PCPs to diagnose and manage asthma, COPD and overlap syndrome patients.	PCP's office, Laboratory facility for conducting spirometry and pulmonary specialist’s office. Communication over internet
Fadaizadeh et al [36]	1. 58 Thoracic surgery patients; 2. consulting physician located in a remote hospital	To assess the advantage of teleICU by accelerating consultations and bringing physicians’ satisfaction from teleconsultation outcomes	1. Comparison of the pace of teleconsultation and regular(bedside) consultation of ICU patients admitted during the year before starting tele-ICU; 2. the physicians’ satisfaction from teleconsultation	Tertiary pulmonology/ thoracic surgery hospital (location of specialist is not mentioned)
Weikert et al [37]	Databased analysis no study population. Data from 239 hospitals located in 46 cities in 12 countries	To support referring centres to interpret HRCT with respect to UIP in patient with suspected Idiopathic Pulmonary Fibrosis (IPF)	Feasibility of cross-border teleradiology for the provision of state-of-the-art reporting	Referral hospitals (location of specialist is not mentioned)
Wrenn et al [29]	86 Primary care providers (PCP) from eight adult primary care sites at the University of California, San Francisco (UCSF), USA, 195 patients	To analyse how the teleconsultation program affects clinical management of patients in primary care	Percentage of specialist recommendations PCPs implemented, and the proportion of patients with a specialist visit in the same specialty as the teleconsultation, emergency department visit, or hospital admission during the subsequent six months	PCPs office (location of specialist is not mentioned)

#### Reliability

The use of specialist to non-specialist teleconsultation was considered reliable in terms of technical feasibility [28,29,33,35,37]. However, concerns were raised in one study that the pulmonary specialist relied primarily on medical history, medical data from tests including radiology, and a limited physical exam performed by a trained nurse or respiratory therapist to arrive at clinical diagnosis [28].

#### Improved patient access

The systems utilised in multiple studies were reported as improving health care access to patients. Referred patients were able to receive medical subspecialty care closer to home, obviating the need for long-distance travel to receive an in-person medical consultation [28,33,35] (**Table 3**).

#### Use of teleconsultation for diagnosis

Three papers primarily focused on disease diagnosis [31,32,35]. The Italian Alliance study shows that telespirometry in the primary care setting could reliably demonstrate spirometric abnormalities and detect airflow limitation even in asymptomatic patients who are at risk of suffering from CRDs [31,32]. Another study also supported the finding that teleconsultation was feasible, effective and efficient in supporting PCPs to diagnose and manage asthma, COPD and overlap syndrome patients [35] (**Table 3**).

#### Acceptability

Specialist to non-specialist teleconsultation is highly dependent on the collaboration between health care providers and how acceptable they found the systems. Fadaizadeh et al. reported high physician satisfaction and acceptance of teleconsultation by the specialists [36]. Further, telespirometry was well accepted and could be easily performed by a large number of PCPs [31,32]. The rate of acceptable spirometric tests can be improved if the tests are performed under the supervision of trained technicians rather than by the PCPs [28,33,35]. One study showed that the service was perceived to stimulate cooperation between primary and secondary care, and deliver support to patients locally which is important in rural areas [35]. Telepulmonology, by improving the collaboration between PCP and pulmonary specialist, may prevent unnecessary face-to-face referrals thus saving time, and aiding in preventing under and misdiagnosis of COPD [34].

### Disease conditions

COPD was addressed in two studies [30,34], both asthma and COPD in three studies [31,32,35], one study focused on general respiratory conditions [28] and two studies on multiple specialities, pulmonology being one of them [29,33]. Fadaizadeh et al. specifically investigated patients who had undergone thoracic surgery with multiple organ failure [36] and Weikert et al. included patients with presence of Usual Interstitial Pneumonia (UIP)-pattern in patients with suspected Idiopathic pulmonary fibrosis (IPF) [37].

### Study designs

The 10 papers included in this review are summarised in **Table 2** and the aims and outcome measures in the included studies are described in **Table 4**. There were no randomised controlled trials or controlled before-and-after studies. Four studies, which yielded five papers, were cross-sectional studies which described numbers of patients and health systems where teleconsultations were implemented [28,31,32,34,36]. Two of these five publications were about ongoing systems described retrospectively [28,34]. Three papers described the ongoing systems [29,33,37], two of which used descriptive statistics to report the outcomes quantitatively [29,37] and the remaining one was a narrative technical description of the teleconsultation system [33]. Only one study, which described three case reports, did not include any quantitative analysis [30] and one study described data at baseline and assessed the patients' clinical outcomes longitudinally after the intervention [35].

### Country of the studies

All but one of the included studies were conducted in high-income countries. Five papers referred to four studies conducted in Europe [31-35], two in the US [28,29] and one each in Japan [30] and Iran [36]. One study was conducted in a network collaboration of 12 hospitals in Central Eastern Europe and Asia [37] (**Table 2**).

### Quality assessment and limitations of the included studies

The quality assessment of the studies was done using JBI tools [27] (**Table 5****,** **Table 6** and **Table 7**). All papers were rated as moderate or low quality. Papers were assessed based on study types. Three papers were assessed as text or opinion papers (**Table 5**), half of the selected papers were analytical cross-sectional studies (**Table 6**), one a study of diagnostic accuracy and one a case study (**Table 7**) and were assessed using respective parameters. All the selected studies have certain limitations (**Table 3**). The opinion papers [29,33,37] did not logically defend incongruence with the literature/sources, whereas the confounding factors were either not considered or mentioned in the cross-sectional analytical studies [28,31,32,34-36]. The one case study [30] reported is a low-quality paper.

**Table 5:** Quality assessment of the selected publications using the JBI tools for critical appraisal (text & opinion papers)*

Study title	1. Is the source of the opinion clearly identified?	2. Does the source of opinion have standing in the field of expertise?	3. Are the interests of the relevant population the central focus of the opinion?	4. Is the stated position the result of an analytical process, and is there logic in the opinion expressed?	5. Is there reference to the extant literature?	6. Is any incongruence with the literature/sources logically defended?
Bernocchi et al [33]	1	3	1	1	3	3
Weikert et al [37]	1	1	1	1	2	2
Wrenn et al [29]	1	3	1	1	1	3

JBI – Joanna Briggs Institute

*1 = Yes, 2 = No, 3 = Unclear, 4 = not applicable.

**Table 6:** Quality assessment of the selected publications using the JBI tools for critical appraisal (analytical cross-sectional study)*

Study title	1. Were the criteria for inclusion in the sample clearly defined?	2. Were the study subjects and the setting described in detail?	3. Was the exposure measured in a valid and reliable way?	4. Were objective, standard criteria used for measurement of the condition?	5. Were confounding factors identified?	6. Were strategies to deal with confounding factors stated?	7. Were the outcomes measured in a valid and reliable way?	8. Was appropriate statistical analysis used?
Raza et al [28]	1	1	1	1	2	2	1	1
Averame et al [31]	1	2	1	1	3	3	1	1
Bonavia et al [32]	1	1	2	1	3	3	2	3
Thijssing et al [34]	1	1	1	1	3	2	1	1
Fadaizadeh et al [36]	1	1	1	1	2	2	1	1
Metting et al [35]	1	1	3	1	2	2	3	1

*1 = Yes, 2 = No, 3 = Unclear, 4 = not applicable.

**Table 7:** Quality assessment of the selected publications using the JBI tools for critical appraisal (case report)*

Study title	1. Were patient’s demographic characteristics clearly described?	2. Was the patient’s history clearly described and presented as a timeline?	3. Was the current clinical condition of the patient on presentation clearly described?	4. Were diagnostic tests or assessment methods and the results clearly described?	5. Was the intervention(s) or treatment procedure(s) clearly described?	6. Was the post-intervention clinical condition clearly described?	7. Were adverse events (harms) or unanticipated events identified and described?	8. Does the case report provide takeaway lessons?
Koizumi et al [30]	1	2	1	3	2	3	1	1

*1 = Yes, 2 = No, 3 = Unclear, 4 = not applicable.

## CONCLUSIONS

We found no controlled trials and relatively few papers mainly of a moderate or low quality which explored specialist to non-specialist teleconsultation in the management of CRDs. This is in contrast to other specialities where comparatively more papers are available describing doctor to nurse or specialist to non-specialist consultation including dermatology [38], psychiatry [39,40] and radiology [41].

Of the included papers a few described systems which did not focus on any one particular disease and lung diseases were just one of the conditions which were managed using these teleconsultation systems. Although some studies indicated that such specialist to non-specialist consultation may have resulted in more local treatment and a lower proportion of referred cases to specialists. The absence of baseline data in some means it is not clear to what extent it may have increased appropriate referrals for patients who previously did not have easy access to diagnosis.

Teleconsultation in the PCPs office with the remotely situated consultant in the hub and spoke model was the most preferred way of conducting specialists to non-specialist consultation and the audio mode was primarily used for consultation. This concludes to be one of the most logistically feasible models for PCPs and specialists and also fulfils the patients’ requirements. Very few studies were conducted in the LMICs and most were reported from European counties. It is possible that specialist to non-specialist consultations for CRD diagnosis and management using teleconsultation are taking place but are not being reported in academic literature. The studies we selected were relatively recent and published during the last decade.

While the available literature does not indicate any generalizability, it shows some encouragement that specialist to non-specialist teleconsultation may facilitate diagnosis and management of CRDs to the benefit of patients. However, CRD diagnosis using specialist to non-specialist teleconsultation was more pragmatic and manageable as against the CRD management.

The use of teleconsultation may potentially help patients for CRD diagnosis and management, where face-to-face consultations are not available [42]. Studies have proven to have a positive impact on the quality of life of the patients suffering from CRDs and acceptance by the health care provider [43]. Further, high-quality research to demonstrate the efficacy of the use of specialist to non-specialist consultation for managing CRDs is required to be conducted in controlled settings. A strategy to build evidence to deploy specialist to non-specialist teleconsultation to manage CRDs will aid further research [44,45]. More high-quality controlled studies are required to confirm these suggestions.

## Additional material

Online Supplementary Document
