# Supplementary material for: Specialist to non-specialist teleconsultations in chronic respiratory disease management: A systematic review
Source: J Glob Health. 2021 Mar 27;11:04019. doi: 10.7189/jogh.11.04019 (PMC8294828; doi:10.7189/jogh.11.04019)
Supplement: Online Supplementary Document [file jogh-11-04019-s001.pdf]

Specialist to non-specialist telehealthcare consultations in Chronic Respiratory Disease management: A systematic review

Annex S1

Database: Embase <1974 to 2019 Week 47>
Search Strategy:
----
1 telemedicine/ or telepulmonology/ or telehealthcare/ (22075)
2 Telemedicine/ (22071)
3 Telehealth.mp. (8790)
4 ehealth.mp. (3497)
5 tele*.mp. (250529)
6 telediagnosis.mp. or Remote Consultation/ (8903)
7 electronic consult*/ or teleconsult*.mp. (9635)
8 Videoconferencing/ (3560)
9 econsult.mp. [mp=title, abstract, heading word, drug trade name, original title, device manufacturer, drug manufacturer, device trade name, keyword, floating subheading word, candidate term word] (135)
10 remote diagnosis.mp. (251)
11 face to face.mp. (34756)
12 virtual/ or online consultation.mp. (169)
13 electronic referral/ or ereferral.mp. (45)
14 e diagnosis.mp. (63)
15 1 or 2 or 3 or 4 or 5 or 6 or 7 or 8 or 9 or 10 or 11 or 12 or 13 or 14 (281938)
16 respiratory tract diseases/ or bronchial diseases/ or ciliary motility disorders/ or lung diseases/ or hepatopulmonary syndrome/ or lung abscess/ or lung diseases, interstitial/ or lung diseases, obstructive/ or asthma/ or bronchitis, chronic/ or pulmonary disease, chronic obstructive/ or pulmonary emphysema/ (367206)
17 Pulmonary Disease, Chronic Obstructive/ or Chronic Obstructive Pulmonary*.mp. (93626)
18 Asthma/ (221535)
19 pulmonary*.mp. (716345)
20 Chronic Respiratory Dis*.mp. (5037)
21 16 or 17 or 18 or 19 or 20 (990650)
22 doctor to doctor.mp. (294)
23 physician to physician.mp. (826)
24 expert to non-expert.mp. (187)

25 doctor to nurse.mp. (6556)
26 physician to nurse.mp. (2801)
27 General Practice/ or General Practitioners/ or outpatient.mp. [mp=title, abstract, heading word, drug trade name, original title, device manufacturer, drug manufacturer, device trade name, keyword, floating subheading word, candidate term word] (433301)
28 subspecialist/ or specialist.mp. (145118)
29 physicians, primary care/ or pulmonologists/ (97770)
30 chest physician.mp. (357)
31 22 or 23 or 24 or 25 or 26 or 27 or 28 or 29 or 30 (571653)
32 15 and 21 and 31 (1043)
****

Database: Ovid MEDLINE(R) and In-Process & Other Non-Indexed Citations <1946 to November 22, 2019>
Search Strategy:
----
1 telemedicine/ or telepulmonology/ or telehealthcare/ (20667)
2 Telemedicine/ (20667)
3 Telehealth.mp. (4075)
4 ehealth.mp. (3225)
5 tele*.mp. (184245)
6 telediagnosis.mp. or Remote Consultation/ (4723)
7 electronic consult*/ or teleconsult*.mp. (1123)
8 Videoconferencing/ (1396)
9 econsult.mp. [mp=title, abstract, original title, name of substance word, subject heading word, floating sub-heading word, keyword heading word, organism supplementary concept word, protocol supplementary concept word, rare disease supplementary concept word, unique identifier, synonyms] (83)
10 remote diagnosis.mp. (181)
11 face to face.mp. (24822)
12 virtual/ or online consultation.mp. (84)
13 electronic referral/ or ereferral.mp. (33)
14 e diagnosis.mp. (32)
15 1 or 2 or 3 or 4 or 5 or 6 or 7 or 8 or 9 or 10 or 11 or 12 or 13 or 14 (208494)
16 respiratory tract diseases/ or bronchial diseases/ or ciliary motility disorders/ or lung diseases/ or hepatopulmonary syndrome/ or lung abscess/ or lung diseases, interstitial/ or lung diseases, obstructive/ or asthma/ or bronchitis, chronic/ or pulmonary disease, chronic obstructive/ or pulmonary emphysema/ (289154)

17 Pulmonary Disease, Chronic Obstructive/ or Chronic Obstructive Pulmonary*.mp. (59596)
18 Asthma/ (122583)
19 pulmonary*.mp. (658016)
20 Chronic Respiratory Dis*.mp. (3432)
21 16 or 17 or 18 or 19 or 20 (830749)
22 doctor to doctor.mp. (221)
23 physician to physician.mp. (544)
24 expert to non-expert.mp. (135)
25 doctor to nurse.mp. (912)
26 physician to nurse.mp. (3946)
27 General Practice/ or General Practitioners/ or outpatient.mp. [mp=title, abstract, original title, name of substance word, subject heading word, floating sub-heading word, keyword heading word, organism supplementary concept word, protocol supplementary concept word, rare disease supplementary concept word, unique identifier, synonyms] (150011)
28 subspecialist/ or specialist.mp. (52604)
29 physicians, primary care/ or pulmonologists/ (3260)
30 chest physician.mp. (193)
31 22 or 23 or 24 or 25 or 26 or 27 or 28 or 29 or 30 (207709)
32 15 and 21 and 31 (308)

****

PubMed

Search	Query	Items found
#4	Search ((((((((((((((((((("telemedicine") OR "telehealth") OR "ehealth") OR "tele") OR "telediagnosis") OR "remote consultation") OR "teleconsultation") OR "videoconferencing") OR tele opinion) OR "remote diagnosis") OR "face to face") OR "online consultation") OR "e cure") OR "e diagnosis") OR "online consult*") OR "econsult") OR "virtual consult*") OR "electronic consult*"))) AND (((((((("respiratory tract disease") OR "chronic bronchial") OR lung dis*) OR "chronic obstructive") OR "asthma") OR pulmonary dis*) OR "chronic respiratory") OR chronic pulmonary dis*) OR "chronic obstructive pulmonary disease")) AND ((((((((((((("doctor to doctor") OR "physician to physician") OR expert to non-expert) OR doctor to nurse) OR physician to nurse) OR "general practice") OR "general physician") OR "outpatient") OR "general practitioner") OR "subspecialist") OR "specialist") OR "primary care physician") OR "pulmonologist") OR "chest physician")	237

#3	Search (((((((((((("doctor to doctor") OR "physician to physician") OR expert to non-expert) OR doctor to nurse) OR physician to nurse) OR "general practice") OR "general physician") OR "outpatient") OR "general practitioner") OR "subspecialist") OR "specialist") OR "primary care physician") OR "pulmonologist") OR "chest physician"	329418
#2	Search (((((((("respiratory tract disease") OR "chronic bronchial") OR lung dis*) OR "chronic obstructive") OR "asthma") OR pulmonary dis*) OR "chronic respiratory") OR chronic pulmonary dis*) OR "chronic obstructive pulmonary disease"	379281
#1	Search ((((((((((((((((("telemedicine") OR "telehealth") OR "ehealth") OR "tele") OR "telediagnosis") OR "remote consultation") OR "teleconsultation") OR "videoconferencing") OR tele opinion) OR "remote diagnosis") OR "face to face") OR "online consultation") OR "e cure") OR "e diagnosis") OR "online consult*") OR "econsult") OR "virtual consult*") OR "electronic consult*")	61441

Database: Global Health

Search Strategy:

----

1 telemedicine/ or telepathology/ or teleradiology/ (1165)

2 Telemedicine/ (1165)

3 Telehealth.mp. (369)

4 ehealth.mp. (317)

5 tele*.mp. (18920)

6 telediagnosis.mp. or Remote Consultation/ (8)

7 teleconsultation.mp. (45)

8 Videoconferencing/ (0)

9 tele opinion.mp. (0)

10 remote diagnosis.mp. (20)

11 face to face.mp. (6856)

12 online consultation.mp. (26)

13 e cure.mp. (0)

14 e diagnosis.mp. (6)

15 1 or 2 or 3 or 4 or 5 or 6 or 7 or 8 or 9 or 10 or 11 or 12 or 13 or 14 (25365)

16 respiratory tract diseases/ or bronchial diseases/ or ciliary motility disorders/ or lung diseases/ or hepatopulmonary syndrome/ or lung abscess/ or lung diseases, interstitial/ or lung diseases, obstructive/ or asthma/ or bronchitis, chronic/ or pulmonary disease, chronic obstructive/ or pulmonary emphysema/ (59186)

17 Pulmonary Disease, Chronic Obstructive/ or Chronic Obstructive Pulmonary*.mp. (6792)

18 Asthma/ (15113)

19 pulmonary dis*.mp. (8635)

20 Chronic Respiratory Dis*.mp. (632)
21 16 or 17 or 18 or 19 or 20 (62321)
22 doctor to doctor.mp. (14)
23 physician to physician.mp. (33)
24 expert to non-expert.mp. (11)
25 doctor to nurse.mp. (159)
26 physician to nurse.mp. (134)
27 General Practice/ or General Practitioners/ (3126)
28 specialist.mp. (5317)
29 physicians, primary care/ or pulmonologists/ (0)
30 chest physician.mp. (12)
31 22 or 23 or 24 or 25 or 26 or 27 or 28 or 29 or 30 (8570)
32 15 and 21 and 31 (20)

****
